# De-SUMOylation of FOXC2 by SENP3 promotes the epithelial-mesenchymal transition in gastric cancer cells

**DOI:** 10.18632/oncotarget.2197

**Published:** 2014-07-09

**Authors:** Yan-hua Ren, Ke-jia Liu, Ming Wang, Ya-nan Yu, Kai Yang, Qin Chen, Bin Yu, Wei Wang, Qi-wei Li, Jian Wang, Zhao-yuan Hou, Jing-yuan Fang, Edward T. Yeh, Jie Yang, Jing Yi

**Affiliations:** ^1^ Department of Biochemistry and Molecular Cell Biology, Shanghai Key Laboratory of Tumor Microenvironment and Inflammation, Institutes of Medical Sciences, Shanghai Jiao Tong University School of Medicine, Shanghai, China; ^2^ Institute of Neuroscience, Wenzhou Medical University, School of Medicine, Zhejiang, China; ^3^ Department of Gastroenterology and Hepatology, Ren Ji Hospital, Shanghai Jiao Tong University School of Medicine, Shanghai, China; ^4^ Department of Biliary- Pancreatic Surgery, Ren Ji Hospital, Shanghai Jiao Tong University School of Medicine, Shanghai, China; ^5^ Department of Cardiology, University of Texas MD Anderson Cancer Center, Houston, Texas, USA

**Keywords:** epithelial-mesenchymal transition (EMT), reactive oxygen species (ROS), SENP3, SUMO2/3, FOXC2, gastric cancer

## Abstract

The impact of cellular oxidative stress in promoting the epithelial-mesenchymal transition (EMT) has been noticed. Our previous study shows that SENP3, a redox-sensitive SUMO2/3-specific protease, accumulates in a variety of cancers, but whether SENP3 and SUMOylation involve in the regulation of EMT is unclear. The present study uncovers a novel role of SENP3 in promoting the EMT process in gastric cancer via regulating an EMT-inducing transcription factor, forkhead box C2 (FOXC2). We demonstrate that the expression of mesenchymal marker genes and cell migration ability are enhanced in SENP3-overexpressing gastric cancer cells and attenuated in SENP3-knockdown cells. A nude mouse model and a set of patient's specimens suggest the correlation between SENP3 and gastric cancer metastasis. Biochemical assays identify FOXC2 as a substrate of SENP3. Meanwhile N-cadherin is verified as a target gene of FOXC2, which is transcriptionally activated by a SUMO-less FOXC2. Additionally, reactive oxygen species-induced de-SUMOylation of FOXC2 can be blocked by silencing endogenous SENP3. In conclusion, SENP3, which is increased in gastric cancer cells, potentiates the transcriptional activity of FOXC2 through de-SUMOylation, in favor of the induction of specific mesenchymal gene expression in gastric cancer metastasis.

## INTRODUCTION

The epithelial to mesenchymal transition (EMT) is a process by which cells lose their epithelial phenotype and acquire a mesenchymal one, which is accompanied by a decreased expression of epithelial markers, such as epithelial cadherin (E-cadherin), and an increased expression of mesenchymal markers, such as N-cadherin, fibronectin and vimentin. EMT thus enables cells to migrate to adjacent tissues or distant organs, correlating with cancer invasion and metastasis [1].

The dramatic phenotype shift from epithelia to mesenchyme is based on a profound alteration in gene expression profiles that is controlled by a small number of transcription factors (TFs). The typical EMT-inducing transcription factors, or EMT-inducing TFs, include Snail, Twist, Zeb families among others. Most of them function as transcription repressors for the expression of epithelial marker genes, and some of them, in parallel, as activators for the expression of mesenchymal genes [2]. The extracellular signal molecules that activate these EMT-inducing TFs, such as transforming growth factor-beta (TGF-β), and their downstream signaling cascades, have been intensively studied [3-5]. An increasing number of microRNAs have been recently found as important regulators that target the transcription or translation of these EMT-inducing TFs [6]. At the post-translational level, phosphorylation and ubiquitination are reported to modulate the turnover of the Snail and Twist proteins [7-9]. However, our understanding of the post-translational modifications of EMT-inducing TFs, especially those elicited by specific EMT inducers, is fairly limited.

Mesenchyme forkhead 1 (also known as Forkhead box protein C2, FOXC2) is a transcription factor that has been newly added into the list of EMT-inducing TFs [10, 11]. Its expression can be stimulated by TGF-β as well as other EMT-inducing TFs [11]. But, nothing has been reported about the post-translational modifications of FOXC2 under the EMT context. Its direct target genes for EMT induction have also not been identified.

The role of ROS in EMT has gained much attention during the past years [12-14]. TGF-β is the most prominent EMT-inducer in the cancer microenvironment [4, 14-16]. Upon TGF-β stimulation, a significant of intracellular ROS is increased, which is responsible for the activation of a number of EMT-related signaling pathways [17-19]. ROS are also required in the EMT induced by many other factors [19-22]. MMP-3 induces EMT through pathway dependent upon production of ROS [23]. A recent publication showed that administration of hydrogen peroxide (H_2_O_2_) directly induces the EMT [24]. Given that a redox control of the EMT has become an emerging view, it is necessary to define redox-sensitive proteins that mediate ROS-induced or -dependent EMT.

We have previously found that a sentrin/SUMO2/3-specific protease, SENP3, is a redox-sensitive molecule that accumulates under cellular oxidative stress. Increased SENP3 regulates the functions of the substrates in cancer cells through reversal of SUMO2/3 modification, which leads to the enhanced cell proliferation, tumorigenesis and angiogenesis [25-27]. Our work also reveals an increase of the SENP3 protein in gastric carcinoma and other types of human cancers [25]. In an attempt to determine whether SENP3 contributes to the EMT and gastric cancer metastasis, we performed the present study. Our findings suggest that SENP3 mediates the EMT-inducing effects of ROS through de-SUMOylating a new substrate, FOXC2, and thus, potentiating the function of FOXC2 as a transcriptional activator for the expression of mesenchymal genes.

## RESULTS

### SENP3 expression correlates with the EMT in gastric cancer cells

**Figure 1:**
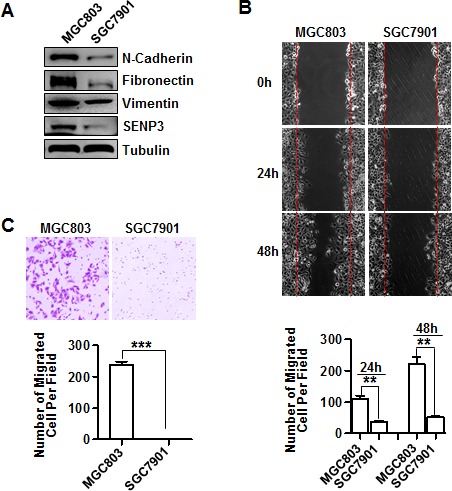
SENP3 expression correlates with the EMT in gastric cancer cells (A) The expressions of EMT marker genes and SENP3 in SGC7901 and MGC803 cells were assessed by immunoblotting. (B) Representative images of wound-healing in SGC7901 and MGC803 cells were shown. The number of migrated cells within the areas of healing surpassing the red lines was calculated, and each experiment was repeated 3 times. **: *P* < 0.01. (C) Representative images of transwell assays (6 hours) in SGC7901 and MGC803 cells were shown. The number of migrated cells on the surfaces of membrane was calculated in 3 fields respectively, and each experiment was repeated 3 times. ***: *P* < 0.001.

We screened the SENP3 protein level in a few available human gastric cell lines, and chose two cell lines with marked differences in the level of SENP3, SGC7901, with barely detectable SENP3, and MGC803, with abundant SENP3, to examine their EMT gene expression and phenotypes. Coincidently, the expression levels of a few typical mesenchymal marker genes, N-cadherin, vimentin, and fibronectin, were significantly lower in SGC7901 cells than in MGC803 cells (Fig. 1A). The typical epithelial marker gene E-cadherin was not detectable in either cell line (data not showed). Scratch wound-healing and transwell assays showed that the cell migration ability of SGC7901 cells was lower than that of MGC803 cells, as evidenced by the quantifications of migrated cell numbers in areas of healing (Fig. 1B) and cell numbers of transwells (Fig. 1C). To exclude that the differences in migrated cell numbers might be contributed by a difference in cell proliferation, we cultured cells in a serum-free medium to slow down cell proliferation. In addition, we compared the whole viable cell numbers and found no differences between two cell lines during the given time of culture (Supplementary Fig. S1). These results suggest that SENP3 expression correlates with EMT in gastric cancer cells.

### SENP3 induces the EMT in gastric cancer cells

**Figure 2:**
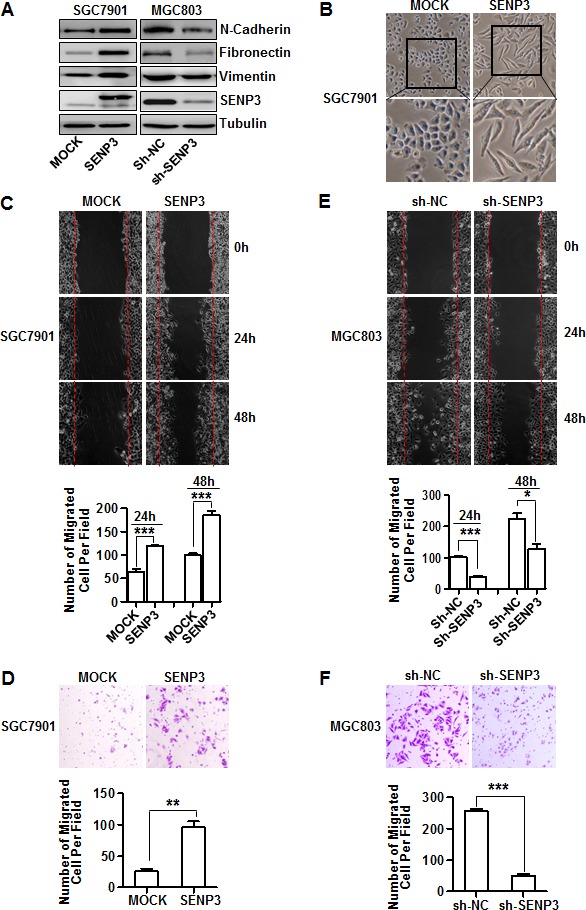
SENP3 induces the EMT in gastric cancer cells (A) The protein levels of EMT markers and SENP3 in the stable cell lines SGC7901-SENP3 (SENP3 overexpression) and MGC803-sh-SENP3 (SENP3 knockdown). (B) Cell morphology of SGC7901 cells with or without SENP3 overexpression. (C, D) Representative images of wound-healing (C) and transwell assays (D) in SGC7901-MOCK and SGC7901-SENP3 cells were shown. (E, F) Representative images of wound-healing (E) and transwell assays (F) in MGC803-sh-NC and MGC803-sh-SENP3 cells were shown. Transwell assays in SGC7901-SENP3 (8 hours) and MGC803-sh-SENP3 (6 hours). The number of migrated cells was calculated as in Figure 1B,C, and each experiment was repeated 3 times. ***: *P* < 0.001, **: *P* < 0.01, *: *P* < 0.05.

To validate whether SENP3 could induce the EMT in gastric cancer, we established two stable cell lines in which SENP3 was over-expressed in one with low basal SENP3 (SGC7901-SENP3) or knocked-down in one with high basal SENP3 (MGC803-sh-SENP3). The expression of EMT markers was increased in SGC7901-SENP3 cells and decreased in MGC803-sh-SENP3 cells, compared with their respective controls (Fig. 2A). SGC7901-SENP3 cells obtained a mesenchymal spindle-like morphology (Fig. 2B). Scratch wound-healing and transwell assays clearly demonstrated the inductive effect of SENP3 on gastric cancer cell migration, because, compared with their respective controls, gastric cancer cells overexpressing SENP3 migrated markedly faster (Fig. 2C,D), while SENP3 knockdown cells migrated slower (Fig. 2E,F), as confirmed by the quantitative analyses. Viable cell number determination excluded that the difference of migration between the SENP3-interferred and non SENP3-interferred cells was partially due to the differences in cell growth rates (Supplementary Fig. S2).

### SENP3 promotes gastric cancer cell metastasis *in vivo*

We next used two nude mouse models to assess the role of SENP3 in cancer cell metastasis *in vivo*.

**Figure 3:**
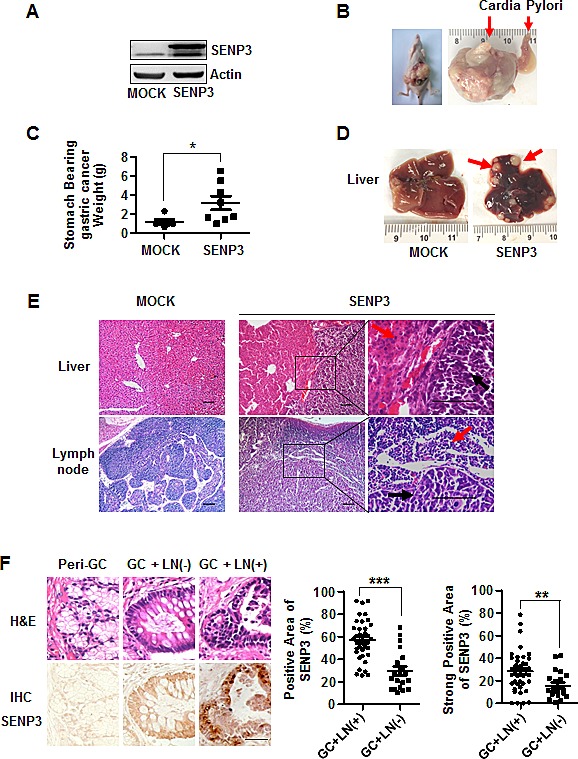
SENP3 promotes gastric cancer cell metastasis *in vivo* (A) The efficiency of SENP3 overexpression in SGC7901-SENP3 cells used for tumorigenesis in nude mice. (B) A mouse (left) and a stomach (right) bearing gastric cancers in the mouse model of orthotopic gastric cancer. (C) The weights of stomach bearing gastric cancer masses in SGC7901-MOCK (n=6) and SGC7901-SENP3 group (n=8).*: *P* < 0.05. (D) Livers from mice with orthotopically implanted gastric cancers. Red arrows indicated metastatic tumors in SGC7901-SENP3 group. (E) Histology of the hepatic tissue (upper) and the lymph node sections (bottom) derived from the representative mice. Black arrows indicated metastasized tumor cells in hepatic tissue (upper) and the lymph node sections (bottom) and red arrows indicated hepatic cells (upper) and lymph cells (bottom). Scale bar=100 μm. (F) The representative fields of patients' tissues of peri-gastric cancer (Peri-GC, n=21) and gastric cancer with or without lymph node metastasis (GC + LN(+), n=39 and GC + LN(-), n=21) that were examined by SENP3 immunohistochemistry (IHC) (bottom) and hematoxylin /eosin (H&E) histology (upper). Scale bar=100 μm. The percentages of the positive area (middle) and strong positive area (right) of SENP3 immunostaining in gastric cancer tissues were measured by using Zeiss KS400 image analyses software. The average areas of SENP3 immunostaining in epithelial cells were obtained from the whole section of each specimen (mean±SEM). ***: *P* < 0.001; **: *P* < 0.01.

An orthotopic gastric cancer metastasis model was established. SGC7901-SENP3 cells (Fig. 3A) were inoculated into nude mice to form tumor xenografts, and the tumors were then recovered, minced into small pieces and implanted beneath the serosa of the stomach of another mouse. Eight weeks later, the tumors grew into large masses in the stomachs (Fig. 3B). The tumors in the SGC7901-SENP3 group were larger than those in the control (Fig. 3C). The livers from each mouse and lymph nodes around the stomach, including pancreaticoduodenal, gastric, caudal mesenteric, jejunal and colic ones, were dissected. The metastasized tumors in the livers were counted under a dissecting microscope. Liver metastases could be found in SGC7901-SENP3 group, but not in the control (Fig. 3D, Table 1). Histological examination demonstrated that the metastasized tumors (Fig. 3E upper, black arrows) grew with the interstitial capsules isolated to the hepatic tissues (red arrows). Lymph nodes metastasis was assessed by histological examination (Fig. 3E, bottom). In many lymph nodes, the metastasized tumors (black arrows) located in the central part, and were relatively isolative of lymphatic tissues (red arrows). The rate of lymph nodes metastasis was markedly higher in the SGC7901-SENP3 group than in the control (Table 2). These data demonstrate that SENP3 promotes gastric cancer cells to metastasize to the liver and surrounding lymph nodes.

**Table 1:** liver metastases in orthotopic gastric cancer metastasis model

Groups/ Items	Livers	Hepatic Metastases	Percentage of Hepatic Metastases
SGC7901-MOCK	7	0	0%
SGC7901-SENP3	8	6	75%

**Table 2:** lymph node metastases in orthotopic gastric cancer metastasis model

Groups/ Items	Lymph Nodes	Lymph Nodes Metastases	Percentage of Lymph Nodes Metastases
SGC7901-MOCK	42	25	59.52%
SGC7901-SENP3	48	45	93.75%

(Lymph nodes include pancreaticoduodenal, gastric, caudal mesenteric, jejunal and colic lymph nodes)

Another injection model was used to verify that SENP3 can promote EMT. B16-F10 mouse melanoma cells stably knocked-down or over-expressing SENP3 (B16-sh-SENP3, B16-SENP3) and the controls were injected into the mice (Supplementary Fig. S3A). B16-sh-SENP3 cells exhibited dramatically reduced lung metastasis, while B16-SENP3 cells showed enhanced lung metastasis, compared with their respective controls (Supplementary Fig. S3B, C).

Next we assessed the levels of SENP3 protein in 60 gastric cancer tissue samples; 39 with lymph node metastasis and 21 without, and the cancer-adjacent normal tissues derived from the latter 21 specimens. Immunohistochemical-positive staining for SENP3 was nearly negative in epithelial cells in the normal tissues of peri-gastric cancers (peri-GC), but was readily visible in epithelium of most of the gastric carcinoma tissues. The primary gastric carcinoma samples with metastasis of peri-gastric lymph nodes (LN) (GC + LN(+)) bore further stronger SENP3 staining, compared with the GC + LN(-) samples (Fig. 3F left). Quantitative image analysis confirmed that both the positive area and strong positive area of SENP3 staining were significantly larger in GC + LN (+) samples than in GC + LN (-) samples (Fig. 3F middle, right). These findings imply a possible role of SENP3 in gastric cancer metastasis.

### SENP3 can de-conjugate SUMO2/3 from the EMT-inducing TF FOXC2

**Figure 4:**
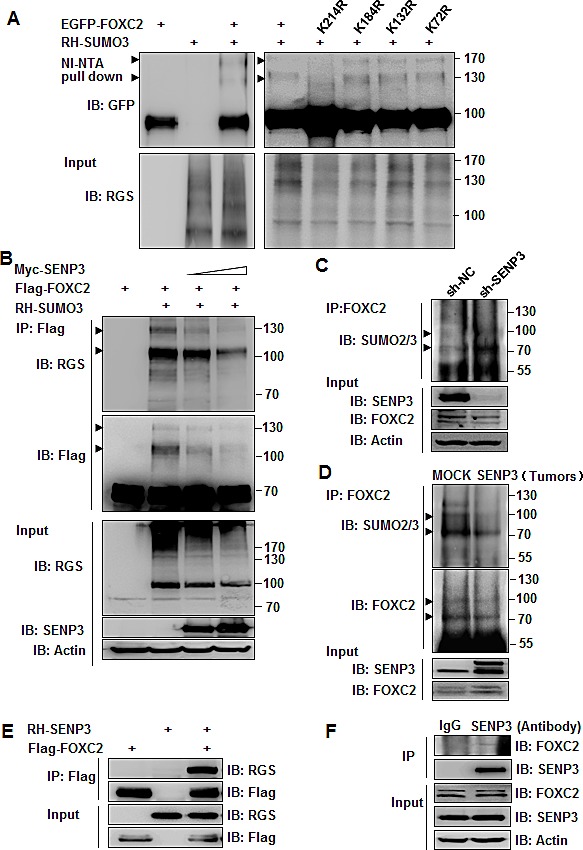
SENP3 can de-conjugate SUMO2/3 from the EMT-inducing TF FOXC2 (A) SUMO3 conjugates of FOXC2, determined by NI-NTA pull down assay in HEK293T cells co-transfected with EGFP-FOXC2 wild-type/mutants and RH-SUMO3. (B) SUMO3 conjugates of FOXC2, determined by Flag immunoprecipitation in HEK293T cells co-transfected with Flag-FOXC2, RH-SUMO3 and SENP3. (C,D) Endogenous SUMO2/3 conjugates of endogenous FOXC2, determined by co-IP in the lysates of HEK293T-sh-SENP3 stable cells (C) and gastric cancers (D). Tumor tissues were collected from two representative orthotopic cancer mice of each group. Arrowheads indicated SUMO3-conjugated FOXC2 in A, B, C and D. (E, F) SENP3-FOXC2 interaction determined exogenously in HEK293T cells co-transfected with two plasmids (E), and endogenously in MGC803 cells (F).

SENP3 is a SUMO2/3 protease that is enriched in the nucleolus and accumulates in the nucleoplasm under oxidative stress, where it interacts with many nuclear proteins. We thus speculated that the effect of SENP3 to induce the EMT might depend on its enzymatic activity to directly de-conjugate SUMO2/3 from EMT-inducing TFs. We predicted the probability of SUMOylation of a few classic or newly identified EMT-inducing TFs, i.e., Snail, Twist, Slug, and FOXC2, using an open software, and found that FOXC2, but not the remaining, had SUMOylation motifs with high probability at K214, K132, K72, and K184 (Supplementary Fig. S4). We constructed plasmids of wild-type FOXC2 or the mutants in which the predicted Lys residues of the SUMOylation sites were replaced by Arg that did not allow SUMOylation. The SUMOylation of FOXC2 was then examined using NI-NTA pull-down assays in HEK293T cells. The result showed that FOXC2 was conjugated by SUMO3 at K214, as the wild-type FOXC2 was pulled-down with SUMO3 conjugates and displayed two prominent bands on the gel (arrowheads in Fig. 4A), and the K214R mutant lacked these bands; but the remaining three mutants maintained these bands (Fig. 4A). We then tested if SENP3 could remove SUMO3 conjugates from FOXC2 using a Flag IP assay. The results showed that SENP3 de-conjugated SUMO3 from FOXC2 in a dose-dependent manner (Fig. 4B). Furthermore, endogenous FOXC2 was immunoprecipitated in SENP3-absent HEK293T cells, and its SUMO2/3 conjugates were heavier than in SENP3-present cells (Fig. 4C). Endogenous SUMO2/3 modifications on endogenous FOXC2 were further analyzed in the tissues obtained from the orthotopic gastric cancers in the mouse model. Tumors derived from SENP3-overexpressed cells had FOXC2 with much fewer SUMO2/3 conjugates (Fig. 4D). The interaction between FOXC2 and SENP3 was demonstrated by co-IP assays in both exogenous (Fig. 4E, HEK293T cells) and endogenous (Fig. 4F, MGC803 cells) settings. These data confirm that SENP3 can remove the SUMO2/3 modification of FOXC2 at K214 via a direct interaction.

### SENP3 upregulates the transcription of N-cadherin through de-SUMOlyation of FOXC2

**Figure 5:**
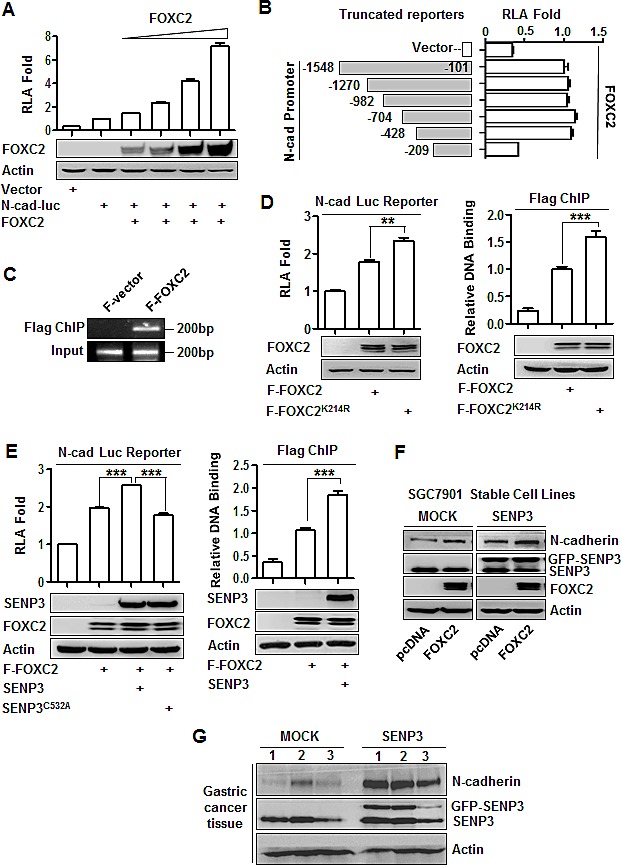
SENP3 upregulates the transcription of N-cadherin through de-SUMOlyation of FOXC2 (A) Luciferase reporter assay in HEK293T cells co-transfected with the vector/N-cadherin promoter reporter and different doses of FOXC2. Relative luciferase activity (RLA) was shown as mean±SEM of three independent experiments. (B) The constructs of a series of N-cadherin promoter reporter with various truncations (left) and luciferase reporter assay (right) in HEK293T cells that were co-transfected with the vector/the reporter truncates and FOXC2. (C) Flag ChIP assay using the antibody against Flag and the primer of the N-cadherin promoter region −428/−209 in HEK293T cells that were transfected with the Flag-tagged vector (F-vector) / Flag-tagged wild-type FOXC2 (F-FOXC2). (D, E) Luciferase reporter assay (left) and Flag ChIP (right) in HEK293T cells that were transfected with F-FOXC2 /SUMO-less mutant of FOXC2 (F-FOXC2^K214R^) (D), or together with wild-type/dominant negative mutant of SENP3 (E). Both assays were repeated three times and the results were shown as mean±SEM. **: *P* < 0.01; ***: *P*<0.001. (F) The N-cadherin protein levels in the stable cell lines SGC7901-MOCK and SGC7901-SENP3 that were transiently transfected with FOXC2. (G) The SENP3 and N-cadherin protein levels in the representative gastric cancer tissue lysates.

Although FOXC2 serves as an EMT-inducing TF, its target genes for mesenchymal induction remain unknown. We noticed that the expressions of fibronectin and N-cadherin were consistently regulated by SENP3 in various gain- or loss-of-function settings. To clarify the functions of FOXC2 in transcriptional regulation of these two genes, we constructed the luciferase reporters fused to the promoter regions of the human fibronectin and N-cadherin genes. The relative luciferase activity (RLA) of the reporter for N-cadherin was increased in a FOXC2 dose-dependent manner (Fig. 5A), indicating that FOXC2 might be the transcription factor of N-cadherin, and that the reporter, namely N-cad luc reporter, was usable. In contrast, the RLA of the reporter for fibronectin was not changed by FOXC2 overexpression (data not showed), suggesting that fibronectin was not regulated by FOXC2, despite that it could be upregulated by SENP3. N-cadherin was then selected as a representative EMT marker gene potentially regulated by SENP3 through FOXC2. To identify the FOXC2-binding elements, we measured the reporter RLA in the presence of FOXC2 based on a series of truncates of this N-cad luc reporter. The results suggested that the sequence between −428 and −209 bp on the N-cadherin promoter contained the essential elements regulated by FOXC2 (Fig. 5B). A ChIP assay was further performed, and the results verified the binding of FOXC2 at the −428 ~ −209 region (Fig. 5C).

To determine the role of the SUMOylation of FOXC2 in the regulation of N-cadherin transcription, the reporter RLA and FOXC2 binding were analyzed in cells overexpressing the FOXC2 mutant lacking the SUMOylation lysine, FOXC2^K214R^. The results showed that N-cadherin transcription could be further enhanced (1.4-fold) by this SUMO-less FOXC2, compared with its wild-type counterpart. The corresponding ChIP assay suggested that the SUMO-less FOXC2 had stronger binding with the N-cadherin promoter (Fig. 5D). The transcriptional activity of FOXC2 was increased by SENP3, not SENP3^C532A^, a dominant-negative form of SENP3. The corresponding ChIP assay also suggested that SENP3 enhanced the binding of FOXC2 with the N-cadherin promoter (Fig. 5E). Furthermore, we examined the N-cadherin protein level in SGC7901 cells overexpressing SENP3 with additional over-expression of FOXC2. The results showed that SENP3 increased N-cadherin expression, which depended on the presence of FOXC2 (Fig. 5F). Immunoblotting assay using the tissue lysates of three representative tumors grown in the nude mouse stomachs showed that the expression of mesenchymal markers was increased in SGC7901-SENP3 cells (Fig. 5G). Collectively, these results indicate that SUMO2/3 conjugation inhibits, but SENP3 enhances, the transcriptional activity of FOXC2 in controlling N-cadherin expression. This action of SENP3 is through de-conjugation of the SUMO2/3 conjugation of FOXC2.

### SENP3 mediates ROS-induced FOXC2 de-SUMOylation

**Figure 6:**
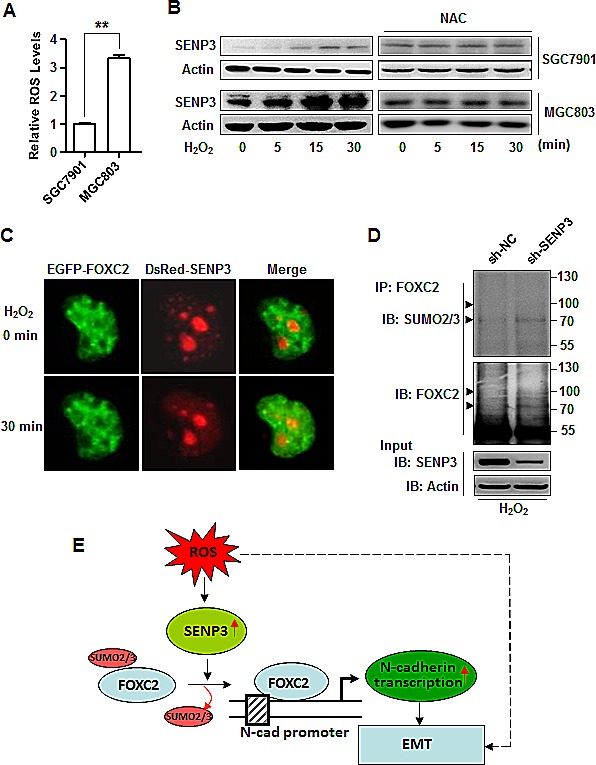
SENP3 mediates ROS-induced FOXC2 de-SUMOylation (A) ROS level determined by DCFH-DA staining and flow cytometric analysis in SGC7901 and MGC803 cells. The results were from three independent experiments. **: *P*<0.01. (B) The increase of SENP3 protein in SGC7901 and MGC803 cells exposed to 200μM H_2_0_2_ in the presence or absence of 5 mM NAC for the indicated time. (C) Live cell imaging of MGC803 cells co-transfected with EGFP-FOXC2 and DsRed-SENP3, and exposed to H_2_O_2_ (200 μM) for 30 minutes. (D) Endogenous SUMO2/3 conjugates of endogenous FOXC2 determined by co-IP in the lysates of MGC803-sh-NC and MGC803-sh-SENP3 cells exposed to 200 μM H_2_O_2_ for 30 min. (E) A model illustrating the molecular mechanism proposed by the present study for the role of SENP3 in the EMT.

Our previous studies have shown that SENP3 is a redox-sensitive molecule. We detected ROS level of the two gastric cancer cell lines, and found that MGC803, which had a higher level of SENP3 (Fig. 1A), also had a higher level of ROS, in contrast to SGC7901 (Fig. 6A). ROS induction of the SENP3 protein was observed when SGC7901 and MGC803 cells were exposed to 200 μM H_2_O_2_ for 5 to 30 min, and this time-dependent SENP3 increase could be blocked by the anti-oxidant N-acetyl cysteine (NAC) (Fig. 6B). Live cell imaging under a time-lapse microscope showed that SENP3, which was normally enriched in the nucleolus and modestly distributed in the nucleoplasm, was markedly increased in the nucleoplasm after the H_2_O_2_ treatment during 30 min, leading to enhanced SENP3-FOXC2 co-localization (Fig. 6C). Moreover, after MGC803-sh-SENP3 cells and the control were exposed to H_2_O_2_, the result of IP showed that ROS-induced decrease of the SUMO2/3 modification of the FOXC2 was dependent on SENP3 (Fig. 6D). These results show that ROS can increase the level of SENP3, co-localization of SENP3 with FOXC2, and de-SUMOylation of FOXC2 in gastric cells. Taken togher, these data suggest that SENP3, which is induced by ROS in gastric cancer cells, may mediate the ROS-induced EMT process. SENP3 de-SUMOylating FOXC2 leads to an enhanced transcriptional activity of FOXC2. Consequently, the mesenchymal marker genes, e.g., N-cadherin, are transcriptionally upregulated (Fig. 6E). This mechanism may explain the role of SENP3 in the metastasis of gastric cancers, although other ROS-dependent EMT-inducing signaling may also be contributive (dash line in Fig. 6E).

## DISCUSSION

FOXC2, previously known to be involved in specifying mesenchymal cell fate during embryogenesis, was identified as an EMT-inducing TF that associates with cancer metastasis by the Weinberg group. They showed that FOXC2 overexpression elicits the expression of mesenchymal marker genes. In contrast with the effects of Snail, Slug, SIP1, Goosecoid and Twist that all strongly reduce E-cadherin expression, the preexisting expression of epithelial cell-specific proteins is only partially reduced by the expression of FOXC2. Hence, FOXC2 is considered to be involved in the induction of the mesenchymal phenotype [10]. FOXC2 was later confirmed to be upregulated in invasive breast cancers [28] and some other metastatic cancers [29, 30] of patients. However, the mesenchymal genes that are transcriptionally controlled by FOXC2, and the post-translational modifications of FOXC2 that regulate its function as an EMT-inducing and cancer-associated TF have not been studied.

SUMOylation is an important modification that usually negatively regulates the activity of transcription factors [31, 32], SUMO2 and -3 are closely related, whereas SUMO1 shares only partial identity with SUMO2 or -3. SUMOylation can be reversed by a family of proteases, named SENPs. Based on our previous studies on SENP3, a SUMO2/3-specific protease, we noticed that the de-conjugation of SUMO2/3 from FOXC2 might serve as a regulatory mechanism for the EMT- inducing effect of FOXC2. During our further investigation, SUMOylation of FOXC2 was reported by Danciu and colleagues [33]. These authors demonstrate that all three SUMO isoforms can be conjugated to FOXC2 in HEK293 cell culture, albeit to different extents: SUMO2 and -3 conjugates are readily detected, whereas SUMO1-modified species are significantly less abundant. They have mapped the site of SUMOylation at the lysine residues 213 and provided evidence that loss of the SUMO enhances the transcriptional activity of FOXC2 in the luciferase reporter as well as in endogenous Hey1 expression in preadipocytes. In their study the SUMO protease SENP2 was used to validate the SUMO conjugate bands. These findings implicate that the loss of FOXC2 SUMO modification could be correlated with FOXC2-associated developmental abnormalities. However, the contribution of SENPs to the regulation of pathological functions of FOXC2, in particular, in the induction of the EMT, has not been examined, and the direct transcriptional target genes of FOXC2 remain unclear [33].

In the present study, we focus on the role of SENP3 in the regulation of FOXC2. We have previously found that SENP3 accumulation in the nucleoplasm allows it to gain a function for controlling various nuclear events in response to stress via regulating a number of nuclear proteins such as p300 and PML [25-27]. FOXC2 is here listed as a novel substrate of SENP3. We highlight this regulation in an EMT process in cancer cells by providing three lines of evidence. First, both exogenous and endogenous SUMO2/3 conjugations on FOXC2 are detectable in gastric cancer cells. Mutagenesis of a plasmid containing human FOXC2 reveals that the lysine residue of SUMOylation is located at 214, which is equivalent to K213 of a mouse FOXC2 that was reported in the work of Danciu et al [33]. Importantly, de-conjugation of SUMO2/3 from FOXC2 by SENP3 exists endogenously in gastric cancer cells. Second, using a series of N-cadherin reporter truncates and ChIP, we confirm a binding of FOXC2 on the N-cadherin promoter, and thus identify N-cadherin as a direct transcriptional target gene of FOXC2. The activity of N-cadhrin reporter is activated by the over-expressed FOXC2 and further increased by a FOXC2 mutant lacking SUMO2/3 modification. Remarkably, the de-SUMOylation of FOXC2 by SENP3 potentiates the function of FOXC2 as a transcriptional activator for N-cadherin expression. Finally, but above all, the role of SENP3 in the metastasis of gastric cancers is strongly supported by the human gastric cancer specimens and the mouse models of metastasis. These findings add novel clues into the role of the SENP family in the EMT because only SENP7 has been previously shown to be contributive [34].

The cancer microenvironment is impacted by redox imbalances of cells; hypoxia, insufficient nutrients, low pH, growth factor /cytokine actions, and immunoreactions such as phagocytosis, can all induce cellular oxidative stress. ROS are thus considered responsible for the initiation, progression, invasion and metastasis of cancers [12]. A plethora of redox-sensitive proteins serves as the links between ROS and malignant cellular phenotypes [35, 36]. Some redox-sensitive transcription factors, e.g., HIF-1, NF-κB and p53, are shown to be important EMT modulators [37-39]. Our present study establishes a direct association between redox-sensitive molecules, a SUMO protease, SENP3, and EMT-inducing transcription factors. Therefore, SENP3 is a mediator that bridges ROS and the EMT. Collectively, these findings uncover a novel oncogenic role of SENP3 and add a layer of regulation to the EMT process at the post-translational modification of FOXC2. Given that gastric cancer is one of the typical oxidative stress-related malignances [40], the present study may increase the understanding toward the molecular mechanism of its pathogenesis.

## MATERIALS AND METHODS

### Cell culture and stable cell lines

The human gastric adenocarcinoma cell lines SGC7901, MGC803 and HEK293T cells were used as in the previous work [41]. B16-F10 cells were purchased from ATCC. To establish the stable cell lines with SENP3 knockdown, shRNAs against SENP3 was constructed with the plvx-shRNA system (Clontech) and introduced to HEK293T, MGC803 and B16-F10 cells. To establish the stable cell lines with SENP3 over-expression, previously used pEGFP-SENP3 plasmid was transfected into SGC7901 and B16-F10 cells with Lipofectamine 2000 (Invitrogen). The positive clones were selected by G418 (Sigma-Aldrich) (0.8mg/ml) and amplified for 14 days, and then sorted by the flow cytometer (Becton Dickson).

### Data of the human gastric cancers

Gastric cancer specimens derived from surgically resected tissues and the related clinicopathological data were obtained in Ren Ji Hospital following an approved protocol, and the patients had not received preoperative chemotherapy or radiation therapy.

### Immunoblotting

Immunoblotting assay was performed as described previously [42].

### Immunohistochemistry (IHC)

The paraffin-embedded sections were subjected to immunohistochemistry as previously described [27]. Quantitative image analysis for the areas of SENP3 immunostaining was conducted using Zeiss KS400 software.

### Cell viability assessment by CCK8

Cell viability was tested using Cell Counting Kit (CCK-8) (Yeasen, China). Cells were cultured in PRMI 1640 (Hyclone) free of fetal bovine serum at 37°C for 8 h, 24 h and 48 h. Then CCK-8 was added to each sample and incubated for 1 h. The absorbance of solution was recorded at 450 nm with a Thermo microplate reader.

### Constructs, mutagenesis and luciferase reporters

DsRed-SENP3 was made by cutting full-length SENP3 from RH-SENP3 and subcloning into BamH I and Xba I sites of DsRed-C1 vectors (Clontech). The GFP-SENP3, Myc-SENP3, RGS-SENP3 and GFP-SENP3^C532A^ were previously used in our work [27].

pEGFP-C1-FOXC2 was made by PCR using specific primers and subcloning the PCR products into BamH I and Xbal І restriction sites of pEGFP-C1. Flag-FOXC2 was made by cutting full-length from pEGFP-C1-FOXC2 and subcloning into BamH I and Xba I sites of Flag-pcDNA vectors (Clontech). pEGFP-C1-FOXC2 Lys/Arg mutant constructs were made by site-directed mutagenesis based on the pEGFP-C1-FOXC2 wild-type construct using a QuikChange mutagenesis Kit (Agilent), as described [27]. The FOXC2 promoter (nucleotides -1548 to -101) was amplified by PCR using the BAC clone (RP11-1017A18, Invitrogen) as the template. The PCR product was inserted into Xho I and Hind III site of the pGL3-basic reporter. Deletions of FOXC2 promoter reporter constructs were established using the method as described previously [27]. The primers used in the constructs were described in supplementary data.

### Scratch wound-healing assay

SGC7901 or MGC803 cells were plated in a 6-well plate. After overnight the monolayer cells were scratched with a pipette tip. The cells were allowed to migrate in medium free of fetal bovine serum until 48 h.

### Transwell migration assay

Transwell assays were performed in 8 μM pore size 24-well transwell plates (BD). The upper chamber was ﬁlled with 2×10^5^ cells in medium without fetal bovine serum. The lower chamber was ﬁlled with medium containing 10% fetal bovine serum as a chemoattractant. After 6 h or 8 h at 37°C, cells on the lower surface of the membrane were ﬁxed with paraformaldehyde and stained with crystal violet. The number of cells migrating was counted under a light microscope (×20, three random ﬁelds per well).

### NI-NTA pull-down assay

The assay followed previous method [27]. Briefly, transfected cells were lysed in a lysis buffer according to the manufacturers' protocols. Ni^2+^-NTA-agarose resin (Qiagen) was then added to the cell lysates and incubated with gentle agitation at 4 °C overnight. The resin was successively washed at room temperature with four different washing buffers. After last washing, RGS-His-tagged proteins were eluted in elution buffer and then subjected to IB. Proteins were detected by various antibodies.

### Flag immunoprecipitation assay

Transfected cells were lysed in a lysis buffer (50 mM Tris HCl, pH 7.4, with 150 mM NaCl, 1 mM EDTA, and 1% TRITON X-100). Flag-beads were added to the cell lysates and incubated at 4 °C overnight. The beads were washed four times in the lysis buffer. After last wash, Flag-tagged proteins were eluted in elution buffer (lysis buffer, cocktail (Roche), 20mM NEM), and then subjected to IB. Proteins were detected by various antibodies.

### Co-immunoprecipitation(co-IP) assay

Co-IP assay to examine endogenous or exogenous SUMO2/3 conjugates of FOXC2 in culture cells and tissues was performed using approaches as previously described [27].

### Luciferase reporter assay

Luciferase reporter assay was performed as described previously [42].

### ChIP assay

HEK293T cells were transfected with plasmids expressing Flag-vector and Flag-FOXC2, and cells were cultured 48 h post-transfection. Protein-DNA was cross-linked in 1%formaldehyde for 10 min at room temperature (RT), then the reaction was quenched by glycine (0.25 M) for 5 min at RT. Cells were harvested and ChIP was performed using a kit (EZ ChIPTM Chromatin Immunoprecipitation Kit, Millipore) according to the manufacturer's instructions. The chromatin samples were sonicated until the size of fragments was between 200bp to 1000bp. Flag-beads were added to the sonicated chromatin samples and incubated at 4 °C overnight. The primer sequences were in primer list. PCR was then undertaken to detect N-cadherin promoter sequences. After denaturation at 98°C for 5 min, 30 cycles were performed using the following PCR program: 98°C for 15 s, 60°C for 15 s, 72°C for 30 s, followed by extension at 72°C for 10 min. Inputs were used as the internal control and were obtained by eluting DNA from 10μl of cell lysates prior to the immunoprecipitation step. ChIP DNA intensity was normalized by input fraction.

### Orthotopic gastric cancer model

Orthotopic gastric cancer model was made according to publication in literature [43]. SGC7901 cells of 1×10^7^ were injected subcutaneously into the 6-wk-old Balb/c nu-nu mice. Two weeks later, tumors were harvested and minced into small pieces (1mm^3^) in RPMI-1640 basal medium. Each tumor piece was placed into a small tissue pocket formed in the middle wall of the greater curvature of the stomach beneath the serosa in another nude mouse, and fixed through purse string suture with 7-0 absorbable sutures. After tumor implantation the stomach was relocated into the abdominal cavity followed by the abdominal closure. All procedures were performed under anesthesia with ketamine (50mg/kg)/ midazolam (10mg/kg) following an approved protocol. Tumor formation and metastasis were analyzed 8 wk later.

### Mouse model of multiple pulmonary melanoma metastases

Transfected B16-F10 cells were established to the stable cell lines, designated as B16-sh-NC, B16-sh-SENP3, B16-SENP3 and B16-MOCK respectively. 1×10^6^ B16-sh-NC or B16-sh-SENP3 and 5×10^5^ B16-SENP3 or B16-MOCK were injected into 6-wk-old BALB/c nude mice via tail vein. Three weeks later tumor formation and metastasis were analyzed.

### ROS detection

2, 7-dichlorodihydrofluorescein diacetate (DCFH-DA; Sigma) was used as ROS capturing reagent in cultured cells with the method described previously [44].

### Time-Lapse Microscopy

MGC803 cells co-transfected with EGFP-FOXC2 and DsRed-SENP3 were plated on a 35 mm glass-bottom dish and exposed to H_2_O_2_. During treatment, cell images were recorded on a Nikon Eclipse Ti inverted microscope. The cell culture environment was maintained at 37°C, 5% CO_2_ and humidified by a chamber. Images were acquired every 5 minutes and finally exported with Nikon Element software (Nikon).

### Statistical Analysis

The statistical significance of differences between groups was assessed using the GraphPad Prism 5 software. The unpaired 2-tailed *t* test was used for the comparison and the level of significance was set at *P* < 0.05.

## SUPPLEMENTAL MATERIAL FIGURES


