# Supplementary material for: De-SUMOylation of FOXC2 by SENP3 promotes the epithelial-mesenchymal transition in gastric cancer cells
Source: Oncotarget. 2014 Jul 9;5(16):7093–104. doi: 10.18632/oncotarget.2197 (PMC4196186; doi:10.18632/oncotarget.2197)
Supplement: Supplementary file 1 [file oncotarget-05-7093-s001.doc]

De-SUMOylation of FOXC2 by SENP3 promotes the epithelial-mesenchymal transition in gastric cancer cells

**Supplemental Material**

**Figure S1: The numbers of viable cells has no difference between MGC803 and SGC7901 cells cultured in medium free of serum.** MGC803 and SGC7901 cells were cultured in medium free of fetal bovine serum for 0 h, 8 h, 24 h, 48 h, and the viable cell numbers were measured by CCK-8.

**Figure S2: The numbers of viable cells has no difference between SENP3-interfered or -non interfered stable cell lines cultured in medium free of serum. (A,B)** SGC7901-MOCK and SGC7901-SEMP3 cells (A), MGC803-sh-NC and MGC803-sh- SENP3 cells were cultured in medium free of fetal bovine serum for 0 h, 8 h, 24 h, 48 h, and the viable cell numbers were measured by CCK-8.

**Figure S3: SENP3 promotes B16 cell metastasis in vivo.** (**A**) The efficiency of SENP3 knocked-down in B16-sh-SENP3 cells and over-expression in B16-SENP3 cells. (**B**) Lungs from B16-sh-SENP3 group (n=6) (bottom) and its control (n=6) (upper). Black dots indicated metastasized tumor colonies and the metastasized tumor colonies were counted under a dissecting microscope. *: *P* < 0.05. (**C**) Lungs from B16-SENP3 group (n=6) (bottom) and its control (n=6) (upper). Black dots indicated metastasized tumor colonies and the metastasized tumor colonies were counted under a dissecting microscope. *: *P* < 0.05.

**Figure S4: SUMOylation sites prediction.** Prediction for SUMOylation probability and sites for EMT-inducing TFs was carried out using an open software SUMOplot™ Analysis Program

**Supplemental Materials and Methods**

| Name of primer Sequence  For pEGFP-C1  FOXC2-f CGCTCTCTCGCTCTCAGGGC  FOXC2-r ATTGTCTGGTTGGGTCGGGG  FOXC2-f TTAGGATCCATGCAGGCGCGCTACT  FOXC2-r CCCTCGAGTCAGTATTTCGTGCAGT  For mutant constructs  K214R-f aaggtggtgatcaggagcgaggcggcg  K214R-r cgccgcctcgctcctgatcaccacctt  K132R-f caacgagtgcttcgtcagggtgccccg  K132R-r cggggcaccctgacgaagcactcgttg  K72R-f gcctaaggacctggtgaggccgcccta  K72R-r tagggcggcctcaccaggtccttaggc  K184R-f agcgggcccacctcagggagccgc  K184R-f gcggctccctgaggtgggcccgct  For FOXC2 promoter  -1548 /-101-f ATCTCGAGTGGGCTGTTCCAGTACATCC  -1548 /-101-r TGAAGCTTA A ACGGCTCCAGGCAGTTTC  -1270 /-101-f ATCTCGAGCCTACCGCGGACCAAAGAT  -1270 /-101-r TGAAGCTTAAAGGCTCCAGGCAGTTTC  -982 /-101-f ATCTCGAGCGGGAGGAATAGGAGAGGG  -982 /-101-r TGAAGCTTAAACGGCTCCAGGCAGTTTC  -704/-101-f ATCTCGAGAGGCCGGAGAACAGTCTCC  -704/-101-r TGAAGCTTAAACGGCTCCAGGCAGTTTC  -428/-101-f ATCTCGAGACATCCTCCACCGGCCAAG  -428/-101-r TGAAGCTTAAACGGCTCCAGGCAGTTTC  -209 /-101-f TCTCGAGGCTATTTGTCATCAGCTCGCTC  -209 /-101-r TGAAGCTTAAACGGCTCCAGGCAGTTTC  -428/-209-f ATCTCGAGACATCCTCCACCGGCCAAG  -428/-209-r CGAGCTGATGACAAATAGCGG |
| --- |

**The primers used in the constructs were listed below.**

**Antibodies**

The following antibodies were used in immunoblotting and Co-IP: N-cadherin, Vimentin, Fibronectin (Epitomics), SENP3 (Santa Cruz), RGS, EGFP, Flag, Tubulin, and β-actin, FOXC2 (Abcam), SUMO2/3 (Cell Signaling Technology). The antibodies against SENP3 (Proteintech Group) was used in immunohistochemistry, and against FOXC2 (Novus Biologicals) was used in co-IP.

**SUMOylation site prediction**

Prediction for SUMOylation probability and sites were carried out using an open software SUMOplot™ Analysis Program
